# Fatty liver in Pakistani cohort with rheumatoid arthritis

**DOI:** 10.12669/pjms.36.4.1984

**Published:** 2020

**Authors:** Abrar Ahmed Wagan, Abdul Qadir Bhutoo, Daim Khan, Abdul Raheem

**Affiliations:** 1 Dr. Abrar Ahmed Wagan, MBBS, FCPS (Medicine), FCPS (Rheumatology), FACR. Assistant Professor, Department of Medicine, Indus Medical College Tando Mohammad Khan, Pakistan; 2 Dr. Abdul Qadir Bhutto, MBBS, MD (cardiology), Dip Cardiology, Consultant Cardiologist, SMMBMU Larkana, Pakistan; 3 Dr. Daim Khan, MBBS. MRCP (UK), Senior Registrar, Department of Medicine, Central Park Medical College Lahore, Pakistan; 4 Dr. Abdul Raheem, MBBS. Post graduate trainee, Central Park Medical College Lahore, Pakistan

**Keywords:** Fatty liver, RA, FRS, DMARD’s, MeTs

## Abstract

**Objective:**

To determine the frequency of fatty liver (non-alcoholic) disease, Framingham 10-year cardiovascular risk score in rheumatoid arthritis patients.

**Methods:**

This study was conducted from September 1^st^ to March 19, 2019, at Rheumatology OPD, Central Park Medical College Lahore. One hundred ninety two seropositive rheumatoid arthritis patients were recruited. Demographic details were noted, BP, BMI, smoking habits, and waist circumference were noted, then sent to radiology department for ultrasound scan of abdomen by an expert radiologist. On next day 10 ml blood was taken by phlebotomist for lipid profile and fasting blood sugar levels, after availability of results 10-years Framingham cardiovascular risk score was calculated.

**Results:**

Females were (81.3%) mean age of (45.4) years, fatty liver was present in n=39 (20.3%). In positive cases comorbid like metabolic syndrome was present (71.8%), diabetes mellitus (33.3%), hypertension (59%) FRS score (intermediate to high in (33.3%), history of hakeem/desi medication use (51.3%), while on regression analysis all study parameters except DMARD’s had significant association with fatty liver (p<0.05).

**Conclusion:**

Nonalcoholic fatty liver disease is very widely prevalent in rheumatoid arthritis patients. As in general population, it is multifactorial in origin and needs careful monitoring and treatment.

## INTRODUCTION

Rheumatoid arthritis is the most common inflammatory arthritis affecting 0.5 to 1% of general population worldwide.^1^ Nonalcoholic fatty liver disease (NAFLD) is defined as the abnormal accumulation of fats (liver fat>5-10% of liver weight), primarily in the form of triglycerides in those with daily use of alcohol (≤ 20 g ethanol/d), disease spectrum ranges simple steatosis to steato-hepatitis with fibrosis, scarring, ultimately leading to cirrhosis, with worldwide prevalence of (10-40%).^2^,^3^

Liver injury is not considered as major extra-articular feature of RA but abnormal liver tests fluctuating with disease activity, mainly elevated alkaline phosphatase, have been seen in 18 to 50% cases.^4^ Ruderman et al in retrospective study on 188 autopsy cases of RA found, 65% of unselected patients with RA had abnormal liver biopsies one-half having mild portal chronic inflammatory infiltrate of the portal tract and small foci of necrosis, and one in four having fatty liver changes.^5^

Shunshuke Mori et al in their observational study of 846 RA found that 42 patients using methotrexate had persistent elevation of transaminases while ultrasound showed fatty liver disease and histological results revealed nonalcoholic steato-heapatitis as most prevalent pattern of liver injury, there was no significant impact of methotreaxte dose and duration on histological severity.^6^

Systematic review by Salliot and van der Heijde identified increased levels of transaminases (ALT, AST) as the second most frequent adverse effects with methotrexate therapy (20.2%) after gastrointestinal probelms.^7^ I t is found that fatty liver disease (NAFLD), diagnosed either by imaging or biopsy, has been associated with methotrexate therapy since long.^8^-^10^

Rajalingham S, et al, in their observational study found that MTX associated NAFLD prevalence was 4.7% and MTX dose was only independent predictor of MTX associated NAFLD with transaminitis.^11^ Our objective was to determine the frequency of fatty liver (non-alcoholic) disease, Framingham 10-year cardiovascular risk score in rheumatoid arthritis patients

## METHODS

After the approval of Institutional review board, (CPMC/IRB/1725, Dated: 10-09-2018), cross sectional study was conducted at rheumatology division department of medicine central park medical college Lahore. Written and informed consents were taken from each study participant after informing and assuring the confidentiality of their data. Sample size of 192 was calculated by using openepi sample size calculator after inserting 4.7% prevalence of MTX associated NAFLD (at 3% margin of error and (95% CI).

Seropositive patients with history of illness for more than one year and those who never had used alcohol in their life were included. Known cases of sero-negative RA, SLE, MCTD, SSc, SjS, psoriatic arthritis, diagnosed cases of hypothyroidism, primary biliary cirrhosis, cardiac cirrhosis, Wilson disease, hemochromatosis, using lipid lowering agents, acute liver disease, chronic liver diseases like cirrhosis and malignancy, current or past use of antiviral therapy for chronic hepatitis (B,C,) & HIV were excluded.

Demographic parameters were asked in detail like age, disease duration, number of medicine in use ,smoking habits, hakeem/Desi medication use for 6 months or more in past two years, history of blood pressure and medicines in use noted, DM was inquired along with insulin or oral hypoglycemic medicines use, waist circumference was measured by standardized formula, circumference of more than 80cms in females and more 90 cms in males taken as criteria point as per International diabetes federation, Blood pressure was measured after mandatory 5 minutes rest, best of two was noted. All study participants were examined by a consultant physician in detail for clinical stigmata of acute and chronic liver diseases and comorbid conditions.

All study participants underwent abdominal ultrasonography for detection of NAFLD as per radiological criteria, (Bright hepatic echoes, increased hepato-renal echogenicity and vascular blurring of portal or hepatic vein have been classified as unique sonographic features of NAFLD^12^ by an expert sonographer having 10 years’ experience, participant(s) with feature(s) of chronic liver disease were found were excluded ,all study participants were requested to return back on next morning with 14 hours fasting state,5 ml of blood was taken with aseptic techniques by a trained phlebotomist, samples were sent to laboratory for FBS, Lipid profile and liver enzyme levels on COBAS-III machine.

After availability of lab results Metabolic syndrome was defined as per IDF criteria^13^ (1) waist circumference >80cms females and >90 males, (2) HDL levels of <50mg females and <40mg males or on treatment with lipid lowering treatment, (3) TG levels of >150mg (4) diagnosed hypertension or treatment of hypertension, BP>130mmhg or diastolic BP >85mmhg (5) FBS 100 mg /dl or more or on treatment of diabetes mellitus, if anyone have 2 out of five, with waist circumference of more than reference range were labeled as metabolic syndrome, their 10-years FRS cardiovascular risk was calculated by Framingham risk calculator by putting values like age, sex, systolic BP, using antihypertensive Medicines, smoking habits, diabetes, and HDL levels, individuals were categorized into FRS <10% score=mild, 11-20%=moderate risk, risk>20% = severe. Data was stored and analyzed using IBM SPSS version 23.0.

## RESULTS

In this study, prevalence of fatty liver disease (NAFLD) was n=39(20.3%). Female gender was predominant (81.3%), majority belonged to age group 31-40 years (32.3%). Over all use of MTX and other DMARD’s was seen in (72%) cases. Hakeem/desi medications use (29.7%), smokers (14.6%), MeTS (31.8%), Hypertension (31.8%), DM (14.1%), FRS between 10-20 as intermediate risk (11.9%). Table-I. Fig-1.

**Table-I:** Baseline Characteristics of Studied Samples (n= 192).

Characteristics		n	%
Age Group	20 - 30 years	44	22.9
	31 - 40 years	62	32.3
	41 - 50 years	61	31.8
	>50 years	25	13.0
Gender	Female	156	81.3
	Male	36	18.8
Medication Use	MTX	95	49.5
	MTX+HQ	28	14.6
	MTX+HQ+SSZ	44	22.9
	LEF	14	7.3
	CS ALONE	11	5.7
Fatty Liver	Yes	39	20.3
	No	153	79.7
HAKEEM/DESI Medication	Yes	57	29.7
	No	135	70.3
Smoking	Yes	28	14.6
	Ex-Smoker	2	1.0
	No	162	84.4
MeTS	Yes	61	31.8
	No	131	68.2
Hypertension	Yes	61	31.8
	No	131	68.2
DM	Yes	27	14.1
	No	165	85.9
FRS	<10 Low Risk	110	72.8
	10 - 20 Intermediate Risk	18	11.9
	>20 High Risk	23	15.2

**Fig-I:**
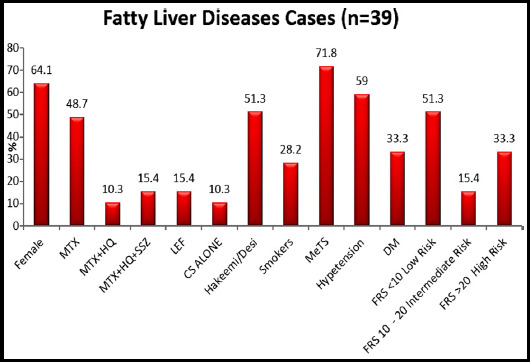
Associated Factors of Fatty Liver Diseases

In RA patients with fatty l ver (n=39) females were n=25 (64.1%), MTX use (48.7%), Hakeem/Desi medication use (51.3%), MeTS (71.8%), hypertension (59%), DM (33.3%), high FRS (33.3%), all other parameters except MTX use had significant association with fatty liver disease (P.05) Table-II.

**Table-II:** Association of Fatty Liver with Studied Parameters.

Factors		Fatty Liver				p-value

		Yes (n=39)		No (n=153)		

		n	%	n	%	
Gender	Female	25	64.1	131	85.6	<0.01*
	Male	14	35.9	22	14.4	
Medication Use	MTX	19	48.7	76	49.7	0.09
	MTX+HQ	4	10.3	24	15.7	
	MTX+HQ+SSZ	6	15.4	38	24.8	
	LEF	6	15.4	8	5.2	
	CS ALONE	4	10.3	7	4.6	
HAKEEM/DESI Medication	Yes	20	51.3	37	24.2	<0.01*
	No	19	48.7	116	75.8	
Smoking	Yes	11	28.2	17	11.1	0.02*
	Ex-Smoker	0	0.0	2	1.3	
	No	28	71.8	134	87.6	
MeTS	Yes	28	71.8	33	21.6	<0.01*
	No	11	28.2	120	78.4	
Hypertension	Yes	23	59.0	38	24.8	<0.01*
	No	16	41.0	115	75.2	
DM	Yes	13	33.3	14	9.2	<0.01*
	No	26	66.7	139	90.8	
FRS	<10 Low Risk	20	51.3	90	80.4	<0.01*
	10 - 20 Intermediate Risk	6	15.4	12	10.7	
	>20 High Risk	13	33.3	10	8.9	

* p<0.05 was considered significant using Pearson Chi Square test.

The mean of different study parameters were, mean age 45.44 (±7.2), mean ALT 83.38 (±35.4), mean AST 71.7 (±29.1), mean HDL 41.8 (±57.8), mean cholesterol 205 (±31.1), mean TG 169.03(±32.5), mean FBS 116.33(±32.5), mean SBP 133.90 (±15.11), mean DBP 85.13 (±7.0) mean, WC 91.33 (±1.5) cms and mean FRS was 16.43 (±17.9). There were significant mean differences obtained between two studied groups with (<p.05). Table-III.

**Table-III:** Mean Comparison of Quantitative Parameters.

Parameters	Total		Fatty Liver				p- value

	(n=192)		Yes (n=39)		No (n=153)		

	Mean	SD	Mean	SD	Mean	SD	
Age (Years)	39.97	10.55	45.44	7.23	38.58	10.82	<0.01*
ALT	46.94	31.69	83.38	35.42	37.65	22.82	<0.01*
AST	41.66	25.21	71.74	29.10	33.99	17.18	<0.01*
ALP	117.27	25.35	123.49	24.70	115.68	25.35	0.08
HDL	48.43	7.88	41.85	7.82	50.10	6.99	<0.01*
Cholesterol	188.09	30.65	205.00	31.17	183.78	29.08	<0.01*
TG	146.45	27.86	169.03	32.57	140.69	23.37	<0.01*
FBS	101.90	22.72	116.33	31.91	98.22	18.07	<0.01*
SBP	126.26	14.77	133.90	15.11	124.31	14.08	<0.01*
DBP	81.82	8.07	85.13	7.02	80.98	8.13	<0.01*
WC	83.07	11.31	91.33	11.55	80.97	10.27	<0.01*
FRS	9.88	13.83	16.43	17.91	7.60	11.32	<0.01*

* p<0.05 was considered significant using Independent sample t-test.

Odds ratio for associated risk factors with fatty liver diseases obtained using binary logistic regression results showed, old age, male gender, high score of FRS, use of Homeopathic medication, smoking, MeTS, hypertension, diabetes and aminotransferases gives significant positive association in cases found with higher risk of fatty liver diseases. Use of methotrexate and different combination of treatments in RA given less association with fatty liver disease. Table-IV. Prevalence of various study metabolic factors in fatty liver positive group is shown in (Bar chart).

**Table-IV:** Estimation of Odds Ratio with 95% confidence interval for Risk of Fatty Liver Disease.

Risk Factors		Odds Ratio (95% C.I)	p-value
Age Group	20 - 30 years	Reference	
	31 - 40 years	10.3(1.2-82.6)	0.02*
	41 - 50 years	18.0(2.3-140.8)	<0.01*
	>50 years	20.2(2.3-174.3)	<0.01*
Gender	Female	Reference	
	Male	3.35(1.5-7.3)	<0.01*
Medication Use	CS ALONE	Reference	
	MTX	0.43(0.11-1.65)	0.22
	MTX+HQ	0.29(0.05-1.47)	0.13
	MTX+HQ+SSZ	0.27(0.06-1.23)	0.93
	LEF	1.31(0.25-6.64)	0.74
FRS	<10 Low Risk	Reference	
	10 - 20 Intermediate Risk	2.25(0.74-6.71)	0.14
	>20 High Risk	5.85(2.24-15.2)	<0.01*
HAKEEM/DESI Medication	Yes	3.30(1.59-6.84)	<0.01*
Smoking	Yes	3.09(1.30-7.32)	0.01*
MeTS	Yes	9.25(4.17-20.53)	<0.01*
Hypertension	Yes	4.35(2.08-9.08)	<0.01*
DM	Yes	4.96(2.09-11.7)	<0.01*
Duration of RA	Yes	1.06(0.99-1.15)	0.08
ALT	Measured	1.04(1.03-1.06)	<0.01*
AST	Measured	1.05(1.04-1.07)	<0.01*
ALP	Measured	1.01(0.99-1.02)	0.09
HDL	Measured	0.84(0.79-0.89)	<0.01*
Cholesterol	Measured	1.02(1.0-1.03)	<0.01*
TG	Measured	1.03(1.02-1.04)	<0.01*
FBS	Measured	1.02(1.01-1.04)	<0.01*
SBP	Measured	1.04(1.01-1.07)	<0.01*
DBP	Measured	1.06(1.02-1.12)	<0.01*
WC	Measured	1.08(1.04-1.12)	<0.01*
FRS	Measured	1.04(1.01-1.07)	<0.01*

* p<0.05 was considered significant for odds ratio.

## DISCUSSION

There is lack of understanding how NAFLD develops in RA patients. Few proposed mechanisms are folate deficiency, high cell turnover, deficiency of purines, pyrimidine thymidine, methionine and accumulation of MTX polyglutamates with genetic polymorphisms in MTX metabolism like C677T polymorphism, increases the chances of its toxicity.^14^-^17^ In our study prevalence of NAFLD was 20.3% (n=39) while in study Rajalingham et al.^11^ it was (4.7%) but MTX and other DMARDs association was not established likewise in our study also.

Shunsuke Mori et al.^6^ and Quintin et al.^18^ in biopsy results of 41 and 32 patients with arthritis on low dose MTX found non-alcoholic steatohepatitis as most frequent lesion, similarly their studies also didn’t established the association of lesions with Methotrexate use.

Ruderman et al.^5^ and Shunshuke et al.^6^ reported that fatty, NASH like lesion or fibrosis exited before the start of MTX introduction. Kuo-Tung Tang et al in their study on chronic hepatitis B and RA patients treated with MTX for 97 months didn’t resulted in development of Cirrhosis.^19^

In a very recent study by Ursini et al found NAFLD prevalence of (25%) and suggested complement factor C3 as potential biomarker for NAFLD.^20^ In a meta-analysis conducted by Young-Ho lee et al about genetic association of NAFLD in RA and psoriatic arthritis found that PPARg Pro12Ala polymorphism is associated with susceptibility to NAFLD in East Asians, but not in European populations.^21^ In Chinese study on general population fatty liver prevalence was (24.7%) with strong association of certain metabolic traits like, higher body fat, obesity, dyslipidemia raised fasting blood sugar levels.^22^

There is well known perception that metabolic syndrome precedes the development of NAFLD and considered as the hepatic manifestation of metabolic syndrome, but A. Lonardo et al, found reverse is true NAFLD precedes the metabolic syndrome, as insulin resistance is present in majority of cases but only minority develops metabolic syndrome, and it has strong association with type 2 diabetes mellitus while these subjects are 1.6 times more likely develop diabetes than NAFLD free cases.^23^ In our study NAFLD positive group (n=39) the prevalence of metabolic syndrome was 71.3% (n=28).

Rheumatic disorders are associated with overexpression of the pro inflammatory cytokine (TNFα), and these disorders respond to treatment with TNF inhibitors (TNFi). TNFα also appears to be a key mediator of fatty liver disease and hepatic fibrosis, so (TNFi) has been proposed as a potential therapeutic target for patients with NASH. Data suggest that TNFi treatment might prevent NAFLD, but there were number of patients with immune-mediated inflammatory diseases to develop NAFLD during treatment with TNFi, which resolved when therapy was stopped.^24^

Sombat Treeprasertsuk et al.^25^ reported that in patients with NAFLD there is higher Framingham cardiovascular risk score than general population, our study results also showed that NAFLD positive group had higher FRS score with mean score of (16.4) while mean score in NAFLD negative group was (9.8), and almost half of the positive group study participants FRS score was in intermediate to high risk scores. Interestingly in our patients with NAFLD there was very high prevalence of hakeem/desi medications use (50%). This is perhaps the first study conducted in south Asia amongst RA patient to look for fatty liver, which has generated local data, along with it we looked at the use of Hakeem/Desi medicine use and presence of metabolic syndrome and calculated CVD risk score as well.

### Limitations of the study

It included cumulative dose of methotrexate couldn’t be calculated as no electronic records of prescribed DMARD’s are available and patients also tend to loose records and miss the doses in between, sample size is small we can’t generalize the study results.

## CONCLUSION

Fatty liver disease not only hampers the ongoing efforts to achieve remission with escalation of conventional dmards and introduction of biological dmards and may enhances the risk of cardiovascular events, which is already high in RA. Regular monitoring with LFT’s and ultrasonography is required at the start of treatment and during follow up.

### Authors Contributions

**AAW:** Conceived, designed, statistical analysis, manuscript writing, editing, take responsibility for integrity of research.

**AQB:** Statistical analysis, editing, manuscript writing, critical review

**DK and AR:** Data collection, manuscript writing, editing.
